# The Usefulness of Posterior Shiny Corner Lesions in the Early Diagnosis of Medial Meniscus Posterior Root Tears

**DOI:** 10.7759/cureus.60605

**Published:** 2024-05-19

**Authors:** Soya Kaneko, Shogo Hashimoto, Akira Honda, Takashi Ohsawa, Ryota Takase, Takeshi Shimada, Hirotaka Chikuda

**Affiliations:** 1 Department of Orthopaedic Surgery, Gunma University Graduate School of Medicine, Maebashi, JPN

**Keywords:** medial meniscus tear, diagnosis, posterior shiny corner lesion, root tear, magnetic resonance imaging

## Abstract

Introduction: Posterior shiny corner lesions (PSCLs) have been reported to be useful for the early diagnosis of medial meniscus posterior root tears (MMPRTs) in surgical patients. However, the usefulness of PSCLs in outpatients, particularly regarding the optimal timing of magnetic resonance imaging (MRI) examinations after injury, remains unknown. We hypothesized that PSCLs would normally be observed in patients with MMPRTs within one month of injury.

Materials and methods: This study included 144 patients with knee pain who visited our hospital between January 2021 and May 2023. MRI findings within and after one month were examined. Fisher's exact test was performed for PSCLs, cleft signs, ghost signs, radial tear signs, bone cysts, and medial meniscus extrusion (MME), which are findings used for the diagnosis of MMPRTs. Time-dependent receiver operating characteristic (ROC) curve analysis was performed for each MRI finding. A binomial logistic regression analysis was performed for age, sex, PSCL, ghost sign, and MME.

Results: PSCLs were observed on 82.6% of the MRI scans within one month, but the positivity rate decreased after one month. After one month, a high percentage of patients had cleft signs and ghost signs. The results of a time-dependent ROC curve analysis showed that the PSCL had better diagnostic ability than the cleft sign, ghost sign, radial tear sign, and MME at a relatively early stage. Additionally, the area under the curve (AUC) of PSCL peaks around 35 days and then declines, reaching 0.8 or less around 40 days. On the other hand, the AUC of the cleft sign and ghost sign began to increase around 30 days after injury, and it exceeded 0.8 after approximately 100 days. The results of the binomial logistic regression analysis revealed significant PSCLs and ghost signs. Independent associations between the PSCL, or ghost sign, and the MMPRT were demonstrated.

Conclusion: This study suggests that PSCLs have a superior diagnostic capability for MMPRT during the early stages of injury compared with other MRI findings in outpatients. In particular, PSCLs have a high positivity rate within one month after injury and a high diagnostic capacity up to 40 days after injury.

## Introduction

The meniscus is a critical structure in the knee joint and is responsible for approximately 40-80% of load transmission [1]. The posterior root of the meniscus is particularly important for maintaining normal biomechanical function [2]. A medial meniscus posterior root tear (MMPRT) disrupts the hoop mechanism of the meniscus, causing extrusion of the meniscus. Consequently, the joint contact pressure increases by 1.3 times [3]. Untreated MMPRTs lead to rapid progression of knee osteoarthritis; 31% of patients require total knee arthroplasty at 30 months, and 87% of patients experience clinical failure at five years [4]. In MMPRT patients with mild osteoarthritis, surgical treatment within three months significantly improved the clinical scores, and high meniscal healing scores at the medial compartment loading area were negatively correlated with the ICRS grade [5]. Thus, the early and accurate diagnosis of MMPRTs remains a clinical challenge.

The diagnosis of MMPRTs relies mainly on MRI findings [6-9]. Highly sensitive diagnostic findings, such as the cleft sign, ghost sign, radial tear sign, and medial meniscus extrusion (MME), have been reported [6-17]. However, in patients with partial damage and those with early-stage MMPRT, these findings may not be clear, making the diagnosis difficult and often resulting in a delayed diagnosis. Recently, a new characteristic finding, the posterior shiny corner lesion (PSCL), has been reported [12,18-21]. PSCLs are bone marrow lesions at the posterior root attachment and are thought to be a characteristic finding in the early stages of injury in MMPRT patients [19,21]. Therefore, PSCL has the potential to aid in the early diagnosis and treatment of MMPRTs.

Existing reports have examined the diagnostic usefulness of PSCL in surgical patients undergoing MMPRTs [18-21].

However, because not all patients undergo surgery, these reports may not be sufficient to demonstrate the usefulness of the PSCL for diagnosing MMPRTs in outpatient settings and the duration of its usefulness after injury.

We hypothesized that PSCLs are closely associated with MMPRTs in outpatients with knee pain, especially within one month after injury, and that PSCLs may, therefore, be an independent predictor of MMPRTs. This study aimed to evaluate the usefulness of PSCLs in the early diagnosis of MMPRTs among outpatients presenting with knee pain and to determine the optimal timing for performing an MRI after injury. We believe that the early diagnosis of MMPRT is, therefore, important since it allows for early therapeutic intervention.

## Materials and methods

Study design

This study was approved by the ethical committee of Gunma University (IRB: HS2023-120). All the methods were performed according to the relevant guidelines and regulations, including the Declaration of Helsinki.

This study included patients who visited our hospital between January 2021 and May 2023 because of knee pain. The following exclusion criteria were applied: age <18 years; neoplastic disease; inflammatory disease; Kellgren-Lawrence (KL) G3 or G4; fracture; no MRI or MRI but no STIR; or uncertain date of injury. MRI findings within one month and after one month were analyzed. We divided patients into groups based on the presence or absence of MMPRT and examined findings that are useful for the early diagnosis of MMPRTs [22-26].

MRI

**Figure 1:**
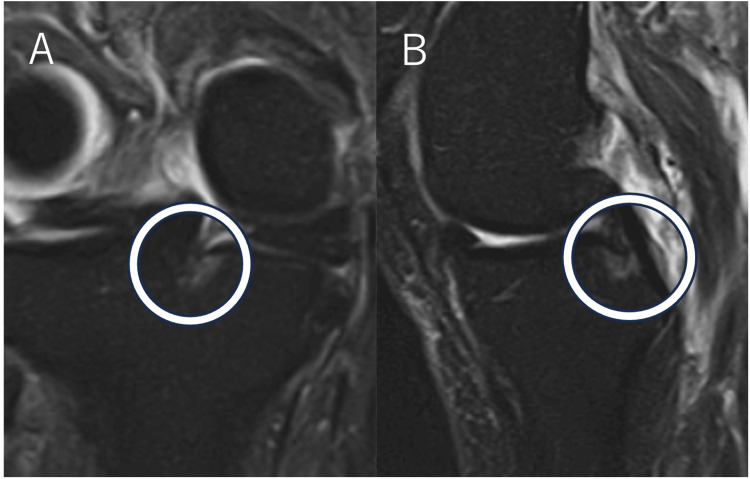
MRI findings of PSCL The STIR (TR 3,300 ms/TE 30 ms, 1.5 Tesla) images are positive findings for PSCL on coronal (A) and sagittal (B) images. MRI: magnetic resonance imaging; STIR: short T1 inversion recovery; PSCL: posterior shiny corner lesion

MRIs were mainly performed at the facility before referral to our hospital. MRI findings within and after one month (PSCL [Figure 1], cleft sign, ghost sign, radial tear sign, bone cyst, MME) [27]. Radiographic findings (femorotibial angle, KL grade, and posterior tibial slope angle) were also examined. Based on a previous report, we determined that the medial meniscus posterior root attachment center was 9.6 (±0.8) mm posterior to and 0.7 (±0.4) mm lateral to the apex of the medial tibial eminence [28]. MMPRT was diagnosed when an MRI showed a tear within 10 mm of the posterior root attachment of the meniscus [5]. Additionally, a T2-weighted fat-suppressed image (TR 3000-4500 ms/TE 30-80 ms, 1.5 Tesla) with a bone marrow lesion at the posterior root attachment of the medial meniscus in coronal and sagittal sections was defined as PSCL-positive [19,21]. Two orthopedic surgeons (S.K. and S.H.) independently assessed the MR images in a blinded manner. Each observer performed each evaluation twice, at least two weeks apart. The reliability of the MRI signs was assessed by examining their interobserver and intraobserver reliability.

Statistical analysis

All statistical analyses were performed using the R programming language. A Fisher’s test was performed for each MRI finding (PSCL, cleft sign, ghost sign, radial tear sign, bone cyst, or MME). The time-dependent receiver operating characteristic (ROC) curve was generated using the R packages “time ROC” and “survival.” A binomial logistic regression analysis using the forced entry method was performed with MMPRT as the dependent variable and age, sex, PSCL, ghost sign, and MME as independent variables. Statistical significance was set at P <0.05.

## Results

Patient characteristics

This study retrospectively evaluated 609 patients. Four hundred sixty-five patients were excluded based on the exclusion criteria. The 465 patients included 55 patients under 18 years of age, 15 patients with neoplastic disease, 205 patients with Kellgren-Lawrence grade 3 or 4, 7 patients with fractures, and 125 patients without MRI, no STIR, or unknown onset.

**Figure 2:**
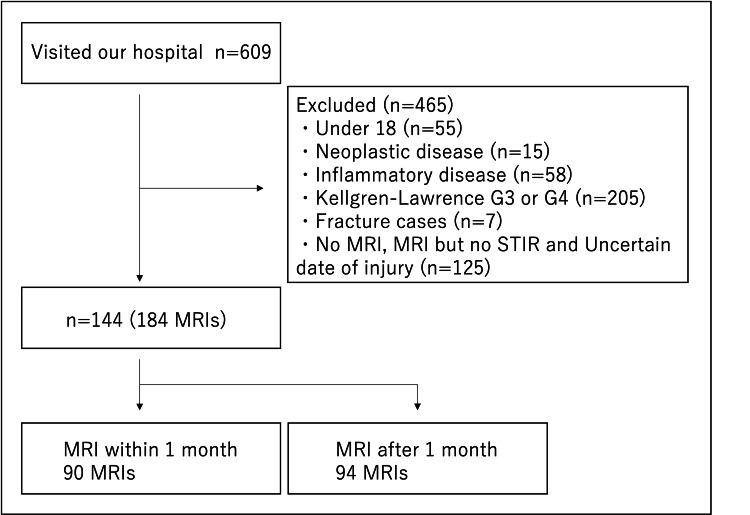
Flow chart MRI: magnetic resonance imaging; STIR: short T1 inversion recovery

Ultimately, the remaining 144 patients (184 MRI scans) were included. MRIs were performed within one month on 90 MRI scans, of which 23 were obtained from patients with MMPRT. There were 94 MRI scans obtained from patients who underwent MRI after one month, 34 of which were from MMPRT patients (Figure 2).

The demographic data of the patients is presented in Table 1. There were six males and 34 females in the MMPRT group, and 49 males and 55 females in the non-MMPRT group. The mean age of patients in the MMPRT group was 62 years. In contrast, in the group without MMPRTs, the mean patient age was 51 years. The other baseline characteristics of the two groups were similar.

**Table 1:** Characteristics of the study group Values are the mean ± standard deviation. MMPRT: medial meniscus posterior root tear; MRI: magnetic resonance imaging

	MMPRT positive group	MMPRT negative group
Number of patients	40	104
Number of MRI scans	60	124
Sex		
Male	6	49
Female	34	55
Age (years)	62±15.7	51±16.1
Height (cm)	156±8.8	163.5±9.2
Body weight (kg)	62.4±15.2	66.4±15.4
Body mass index (kg/m2)	25.5±4.6	24.9±4.7
Duration from injury to MRI (days)	57±93.0	73±94.3
Side		
Right	10	51
Left	30	53
Kellgren-Lawrence grade		
0	0	19
1	18	38
2	22	47
Femoro-tibial angle	176.8±3.7	175±3.7
Posterior sloping angle	9.0±3.1	9.7±3.2

Univariate analysis

MRI findings within one month revealed significant differences in PSCL, cleft sign, ghost sign, radial tear sign, bone cyst, and MME. The PSCL had the highest sensitivity, followed by the ghost and cleft signs. (PSCL, 82.6%; cleft sign, 73.9%; ghost sign, 78.3%; radial tear sign, 52.2%; bone cyst, 17.4%; MME, 39.1%; p<0.05) (Table 2). Compared to these findings, the MRI findings after one month showed significant differences in the cleft sign, ghost sign, radial tear sign, bone cyst, and MME but no significant differences in PSCL. The percentage of patients who tested positive for PSCL decreased significantly to 16.2%. In contrast, the sensitivity of the cleft sign, ghost sign, radial tear sign, bone cyst, and MME significantly increased after one month (cleft sign, 91.9%; ghost sign, 89.2%; radial tear sign, 67.6%; bone cyst, 27.0%; and MME, 64.9%; p<0.05) (Table 3).

**Table 2:** Within one month Fisher’s exact test was used. MMPRT: medial meniscus posterior root tear; MRI: magnetic resonance imaging

	MMPRT positive group	MMPRT negative group	p-value	Sensitivity (%)
Number of MRI scans	23	67		
Posterior shiny corner lesion				
positive	19	4	< .001	82.6
negative	4	63		
Cleft sign				
positive	17	0	< .001	73.9
negative	6	67		
Ghost sign				
positive	18	1	< .001	78.3
negative	5	66		
Radial tear sign				
positive	12	0	< .001	52.2
negative	11	67		
Bone cyst				
positive	4	1	0.014	17.4
negative	19	66		
Medial meniscus extruision				
≥3	10	6	0.002	39.1
<3	13	61		

**Table 3:** After one month Fisher’s exact test was used. MMPRT: medial meniscus posterior root tear; MRI: magnetic resonance imaging

	MMPRT positive group	MMPRT negative group	p-value	Sensitivity(%)
Number of MRI scans	37	57		
Posterior shiny corner lesion				
positive	6	3	0.148	16.2
negative	31	54		
Cleft sign				
positive	34	0	< .001	91.9
negative	3	57		
Ghost sign				
positive	33	0	< .001	89.2
negative	4	57		
Radial tear sign				
positive	25	0	< .001	67.6
negative	12	57		
Bone cyst				
positive	10	3	0.04	27.0
negative	26	54		
Medial meniscus extruision				
≥3	24	10	< .001	64.9
<3	13	47		

The interobserver and intraobserver reliabilities of the assessments were determined by calculating the kappa statistic and were considered satisfactory (kappa value >0.75).

There were a total of seven patients who were PSCL-positive without MMPRTs, four patients who were PSCL-positive within one month, and three who were PSCL-positive after one month. Of the seven cases, three were associated with posterior cruciate ligament (PCL) injuries, three were associated with medial meniscal tears, and one was associated with lateral meniscal tears.

Diagnostic ability of the MMPRTs

**Figure 3:**
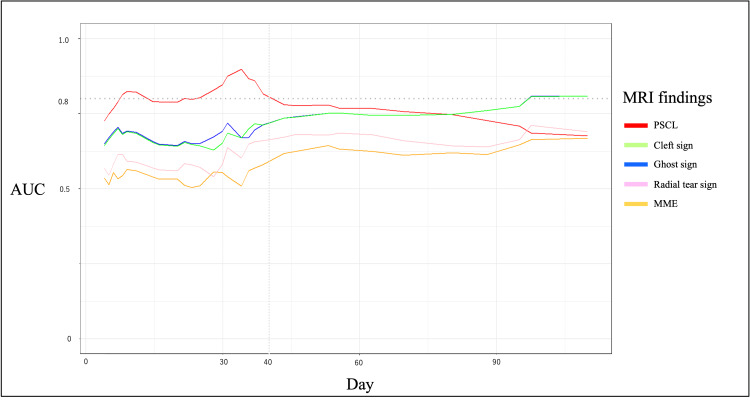
Time-dependent ROC curves The time-dependent ROC curves are a plot of the AUC of each MRI finding over time. An AUC of 0.8 or higher was considered to have sufficient diagnostic ability. ROC: receiver operating characteristic; AUC: area under the curves; MRI: magnetic resonance imaging; PSCL: posterior shiny corner lesion; MME: medial meniscus extrusion

The results of time-dependent ROC curve analysis showed that the PSCL had better diagnostic ability than the cleft sign, ghost sign, radial tear sign, and MME at a relatively early stage (Figure 3). The area under the curve (AUC) of PSCLs peaked at approximately 35 days after injury and thereafter declined below 0.8 after approximately 40 days. On the other hand, the AUC of the cleft sign and ghost sign began to increase around 30 days after injury, and it exceeded 0.8 after approximately 100 days. The AUC of the radial tear sign and MME remained below 0.8 during the entire observation period.

Multivariate analysis

The results of the binomial logistic regression analysis are presented in Table 4. PSCL and ghost signs showed significant independent associations with MMPRT (P<0.05).

**Table 4:** A binomial logistic regression analysis Forced entry method was used.

		95% Confidence Interval (CI)		
	odds ratio (OR)	Minimum	Max	p-value
Age	1.05	0.98	1.12	0.171
Sex	18.00	1.47	220.00	0.024
Posterior shiny corner lesion	102.00	9.15	1130.00	<0.001
Ghost sign	2020.00	81.40	49900.00	<0.001
Medial meniscus extrusion	1.70	0.24	12.20	0.597

## Discussion

The most important finding of this study was that PSCLs had a greater diagnostic capability than other MRI findings, especially in the early stages of injury. The prevalence of PSCLs (82.6%) was higher than that of other findings among outpatients with knee pain whose MRI findings were available within one month. According to the time-dependent ROC curve, PSCLs had the highest diagnostic ability during the early stages, up to approximately 40 days after MMPRT injury. Additionally, the multivariate analysis demonstrated a significant association between PSCL and MMPRTs. PSCL-positive status without MMPRT was associated with medial and lateral meniscal tears and PCL injuries.

PSCLs were first reported as “shiny corner lesions” (SCL) and were found to be associated with meniscal injury [18]. Later, PSCL and the spreading sign were reported in surgical patients in studies that focused only on root findings. A report on the spreading sign suggested that the PSCL is a precursor lesion that develops before other findings. PSCL is thought to be caused by traction force, as reported previously [19,21]. Therefore, the PSCL can be identified early in MMPRT injuries, and because it is caused by a minor external force, it can be assumed that the PSCL disappears early. One possible reason for the higher rate of PSCL positivity in comparison to other findings is that the ghost sign, cleft sign, and radial tear sign are used to evaluate the meniscus body, whereas the PSCL is related to changes in bone bruising. While other findings are more easily detected on MRI as a meniscal tear progresses, the PSCL appears as soon as traction force is applied, even if the meniscal tear has not progressed; thus, the positivity rate is higher in the early phase. We speculated that the reason why the PSCL disappears early is that it is caused by a minor force due to traction force and disappears earlier than bone contusions caused by a major force.

No previous reports have focused exclusively on PSCLs, including outpatients with knee pain, or examined the timing of injury. In this study of outpatients, PSCL was strongly positive in the early stage, and the diagnostic performance of PSCL in the early stage was superior to that of other findings in terms of the time-dependent ROC curve. Logistic regression analysis also revealed an independent and significant association. From a statistical point of view, the results confirmed the usefulness of PSCL in the diagnosis of MMPRTs. Early surgical intervention has been reported to significantly improve clinical scores, and early diagnosis and treatment intervention focusing on PSCL may, therefore, lead to better clinical outcomes.

However, there is a caveat to the use of PSCLs for outpatient diagnosis. It should be noted that PSCLs can be associated with PCL injuries and medial and lateral meniscal tears. These patients are considered PSCL-positive because of the anatomic proximity of the posterior root attachment of the medial and lateral meniscus and the tibial attachment of the PCL, as well as the traction force applied, as in MMPRTs [28-30]. It is important to keep in mind that patients with these conditions can be positive for PSCLs and to combine these findings with other findings to make a diagnosis.

This study has several limitations. First, it was a retrospective study. Second, this study did not include patients with unclear injury dates. In this study, only patients with painful popping and a clear date of injury were included. Patients with painful popping but an unclear date of injury and patients without painful popping were excluded from the study. Third, the MRI conditions were not uniform, and the volume of bone contusions was not measured. Uniform MRI conditions may have yielded different results. Finally, the patient groups within one month and after one month differed. We were not able to compare the same patients, and we were not able to examine the timing of PSCL disappearance.

Regarding clinical relevance, the results of this study may lead to both an earlier diagnosis and the timely treatment of MMPRT, which are expected to improve clinical outcomes.

## Conclusions

This study suggests that PSCLs have a superior diagnostic capability for MMPRT during the early stages of injury compared with other MRI findings in outpatients. In particular, PSCLs have a high positivity rate within one month after injury and a high diagnostic capacity up to 40 days after injury.
